# Staged Full‐Arch Rehabilitation With Tilted and Pterygoid Implants: A Pragmatic Hybrid Analog–Digital Workflow With 2‐Year Follow‐Up

**DOI:** 10.1155/crid/9931993

**Published:** 2026-09-15

**Authors:** Francesco Liborio, Fabio Massimo Sciarra, Emanuele Di Vita, Salvatore Nigliaccio, Davide Alessio Fontana, Pietro Messina, Enzo Maria Cumbo, Giuseppe Alessandro Scardina

**Affiliations:** ^1^ Dipartimento di Medicina di Precisione in Area Medica, Chirurgica e Critica Palermo, Universita degli Studi di Palermo, Palermo, Sicily, Italy

## Abstract

**Background and Aim:**

Full‐arch rehabilitation in patients with advanced maxillary atrophy has evolved considerably through the introduction of tilted and pterygoid implants combined with immediate loading protocols. At the same time, digital technologies have transformed prosthetic workflows. Nevertheless, fully digital procedures may still present limitations in edentulous arches because of scanning accuracy, equipment costs, and restricted accessibility in many clinical settings.

**Case Report:**

A 68‐year‐old male patient affected by terminal dentition and severe generalized periodontitis underwent staged implant‐supported full‐arch rehabilitation. The maxilla was treated first using immediate postextraction placement of six implants, including tilted posterior and pterygoid implants, followed by immediate loading. Six months later, the mandible was rehabilitated using the same treatment philosophy. Prosthetic rehabilitation was performed through a hybrid analog–digital workflow in which conventional implant impressions were integrated with digital scanning and CAD–CAM manufacturing.

**Results:**

All implants obtained adequate primary stability, allowing immediate loading. Clinical and radiographic examinations confirmed successful osseointegration, stable peri‐implant tissues, and absence of biological or mechanical complications throughout the observation period. Definitive CAD–CAM prostheses demonstrated passive fit without requiring intraoral corrections. At 2 years, functional stability, aesthetic integration, and patient satisfaction remained excellent.

**Conclusions:**

This case illustrates that a hybrid analog–digital workflow can represent a practical and predictable alternative for full‐arch rehabilitation in complex implant cases. By combining the precision of conventional impressions with the flexibility of digital prosthetic design, the proposed protocol achieved reliable clinical outcomes while remaining suitable for everyday clinical practice. Although further clinical studies are necessary, this approach may provide a practical and clinically applicable strategy that integrates conventional and fully digital rehabilitation protocols.

## 1. Introduction

Full‐arch rehabilitation of patients with severe maxillary atrophy continues to represent one as of the greatest challenges in implant dentistry, because it requires the simultaneous achievement of mechanical stability, long‐term biological success, and satisfactory aesthetic outcomes. Over the past decades, the introduction of immediate loading protocols together with the placement of tilted and pterygoid implants has considerably expanded the therapeutic possibilities for these patients, frequently allowing clinicians to avoid extensive bone augmentation procedures while reducing treatment duration, surgical morbidity, and overall costs [1–6].

At the same time, the progressive integration of digital technologies has profoundly influenced prosthetic rehabilitation. The widespread use of intraoral scanners, CAD software, and computer‐aided manufacturing has simplified communication between clinicians and dental laboratories, improved patient comfort during impression procedures, and facilitated the management and storage of clinical data throughout treatment [2–6].

Despite these advantages, the application of a completely digital workflow remains challenging in fully edentulous patients. The absence of stable anatomical reference points, together with soft tissue mobility and cumulative stitching errors during image acquisition, may reduce the accuracy of intraoral scans, particularly in long‐span implant‐supported rehabilitations. These limitations can negatively influence the transfer of implant positions and may compromise the passive fit of definitive prosthetic frameworks, occasionally requiring additional clinical adjustments [7–10].

To overcome these limitations, photogrammetric systems have recently been introduced as an alternative method for recording implant positions with high precision. Although encouraging results have been reported, these technologies still require dedicated equipment, specialized operator training, and considerable financial investment. Consequently, their routine implementation remains limited in many clinical environments, particularly where access to advanced digital systems is restricted [11–14].

For this reason, hybrid analog–digital workflows continue to represent a valuable treatment option. By combining the proven reliability of conventional implant impressions with the versatility of digital design and CAD–CAM manufacturing, these protocols seek to preserve prosthetic accuracy while taking advantage of contemporary digital technologies [9, 15, 16].

Although hybrid workflows have already been described in the literature, relatively few reports have focused on their practical application in daily clinical practice, especially when compared with fully digital protocols in terms of accessibility, workflow efficiency, and long‐term clinical performance.

The present case report describes the staged rehabilitation of a patient with terminal dentition using immediate loading of tilted and pterygoid implants through a hybrid analog–digital workflow. In addition to presenting the clinical protocol, this report discusses the rationale behind the selected workflow and its clinical performance after a 2‐year follow‐up period.

## 2. Case Presentation

### 2.1. Patient information

A 68‐year‐old male patient, a heavy smoker with a history of untreated chronic periodontitis, presented complaining of impaired mastication, discomfort during function, and progressive deterioration of the existing prosthetic restorations. Clinical examination, supported by radiographic assessment, revealed terminal dentition in both arches, characterized by generalized periodontal attachment loss and multiple prosthetic failures.

**Figure 1:**
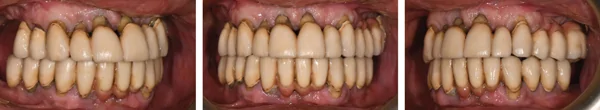
Initial situation of the patient.

The patient′s chief complaint was the progressive loss of masticatory function associated with recurrent prosthetic instability, particularly affecting the maxillary arch. His medical history was otherwise noncontributory, whereas smoking represented the principal systemic risk factor potentially affecting implant therapy and long‐term peri‐implant health (Figure 1).

### 2.2. Clinical Findings

The intraoral examination demonstrated extensive periodontal destruction involving both maxillary and mandibular dentition. Existing full‐arch metal–ceramic prostheses exhibited poor marginal adaptation, generalized mobility, and inadequate periodontal support. Based on the 2018 Classification of Periodontal Diseases, the patient was diagnosed with Stage IV, Grade C periodontitis. Stage IV was assigned considering the presence of interdental clinical attachment loss (CAL) > 5 mm, the loss of more than five teeth because of periodontitis, and the need for complex oral rehabilitation. Grade C was assigned because smoking (> 10 cigarettes/day) represented the principal grade modifier [1].

Owing to the severity of the periodontal condition, the remaining teeth were considered unsuitable for long‐term preservation.

Before treatment, the patient received detailed information regarding the surgical and prosthetic procedures, the importance of meticulous oral hygiene, the necessity of regular maintenance visits, and the potential influence of smoking on implant prognosis. Written informed consent was obtained before the beginning of treatment.

### 2.3. Diagnostic Assessment

A staged rehabilitation protocol was selected. Because the patient′s principal functional complaint involved the maxillary arch, treatment was planned to begin with the maxilla, whereas mandibular rehabilitation was scheduled after completion of the first treatment phase.

Cone beam computed tomography (CBCT) was performed to evaluate residual bone availability and to establish implant positioning.

CBCT examination demonstrated advanced posterior maxillary bone resorption associated with marked maxillary sinus pneumatization, resulting in insufficient bone volume for conventional posterior implant placement. Conversely, the anterior maxilla provided adequate bone availability for implant insertion, whereas the posterior regions required the use of alternative anchorage strategies. In the mandible, sufficient bone volume was available within the interforaminal area, allowing implant‐supported rehabilitation without the need for regenerative procedures.

**Figure 2:**
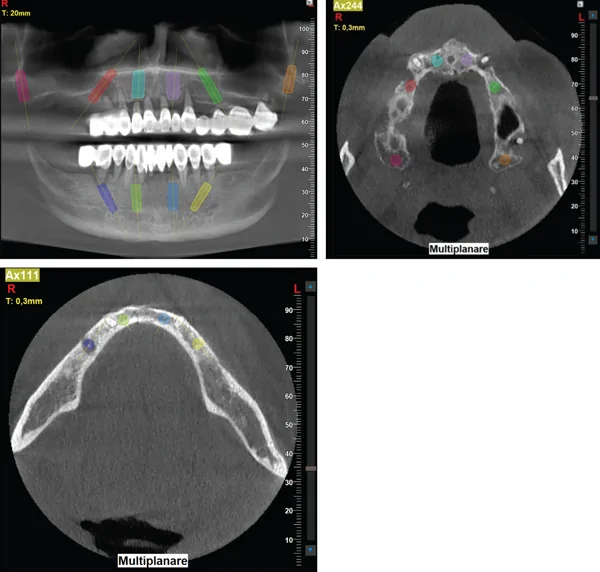
Rehabilitation project on the CBCT DICOM viewer (NewTom NNT).

Based on the clinical and radiographic findings, immediate implant placement with immediate loading was considered feasible. A staged full‐arch rehabilitation using tilted posterior implants and pterygoid implants in the maxilla, followed by mandibular rehabilitation after healing, was therefore selected (Figure 2).

## 3. Therapeutic Intervention

### 3.1. Maxillary Rehabilitation

Following extraction of all remaining maxillary teeth, a full‐thickness mucoperiosteal flap was elevated without vestibular or palatal releasing incisions, extending posteriorly to the tuberosity and pterygomaxillary junction. Implant site preparation was performed freehand under copious saline irrigation according to the preoperative CBCT planning, using anatomical landmarks to guide implant positioning. Parallelism pins were used throughout the procedure to verify symmetry and implant axis orientation.

**Figure 3:**
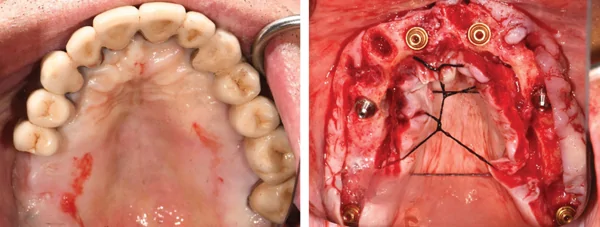
Palatal aspect before surgery and implant positioning.

Six implants (MIS Seven, MIS Implants Technologies, Misgav, Israel) were placed according to the planned prosthetic design. Two axial implants were inserted in the anterior maxilla (4.2 × 11.5 mm), two distally tilted implants were positioned along the anterior sinus wall (3.75 × 16 mm), and two pterygoid implants (4.2 × 13 mm) were inserted distal to the maxillary sinus, engaging the cortical bone of the maxilla, palatine bone, and pterygoid process of the sphenoid. This configuration was selected to maximize posterior support, reduce distal cantilevers, and improve load distribution during immediate function [1, 2, 5] (Figure 3).

The pterygoid implants followed an anteroposterior trajectory with approximately 20°–40° inclination in the sagittal plane and 15°–25° in the lateromedial direction, according to the anatomical characteristics of the pterygomaxillary region and the prosthetic requirements.

Primary stability was achieved for all implants, with insertion torque values ranging between 35 and 50 Ncm, allowing immediate loading. To optimize prosthetic emergence and correct implant angulation, straight multiunit abutments were connected to the axial implants, whereas 30°‐angled multiunit abutments were used on the tilted posterior implants.

### 3.2. Immediate Prosthetic Phase

**Figure 4:**
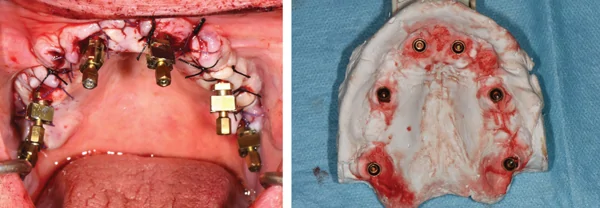
Pick‐up transfers and surgical plaster impression.

**Figure 5:**
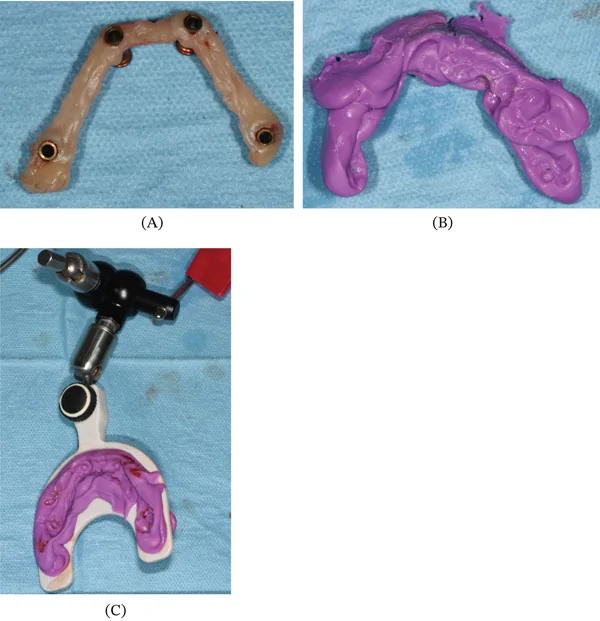
(A) Key jig, (B) occlusal silicone, and (C) facial bow fork.

Immediately after implant placement, definitive implant impressions were obtained using an open‐tray technique with pick‐up transfers splinted by low‐expansion plaster (Figure 4). Maxillomandibular relationships were recorded using rigid silicone registration material, and a facebow transfer was performed to accurately reproduce the spatial relationship between the maxilla and the articulator (Figure 5).

The definitive master cast was mounted on a semiadjustable articulator. The opposing mandibular arch, previously digitized through intraoral scanning and reproduced by three‐dimensional printing, was positioned using the recorded interocclusal relationship.

**Figure 6:**
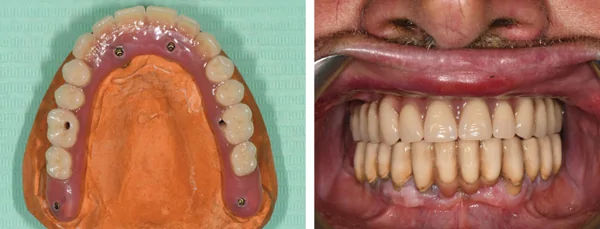
Immediate loading prosthesis on model and delivered.

**Figure 7:**
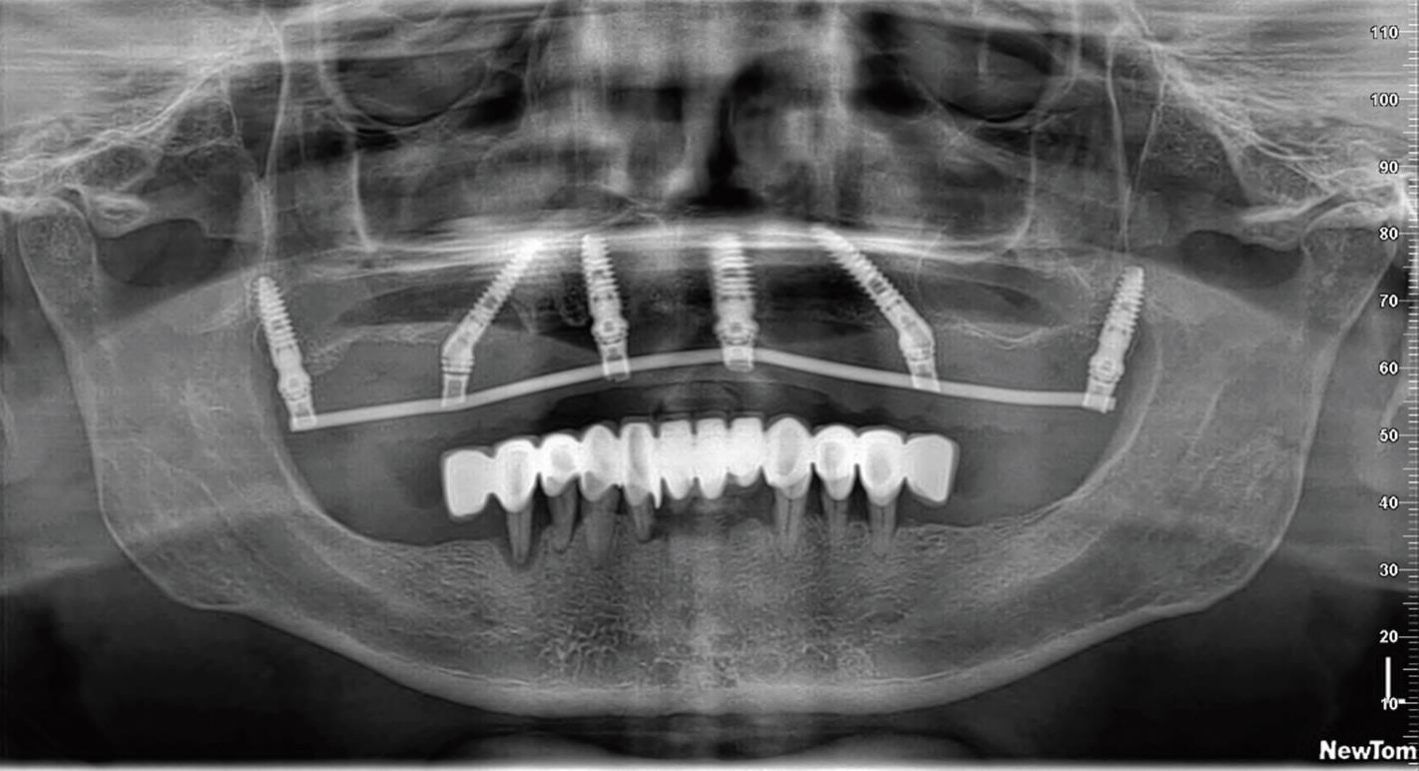
Control OPT after provisional delivery.

This analog workflow allowed fabrication of a screw‐retained immediate provisional prosthesis with accurate fit and occlusion, which was delivered within 24 h after surgery (Figure 6). Postoperative radiographic control confirmed the planned implant configuration (Figure 7). The prosthetic design aimed to minimize distal cantilevers and establish a mutually protected occlusal scheme with group function during lateral excursions.

### 3.3. Definitive Maxillary Prosthesis

**Figure 8:**
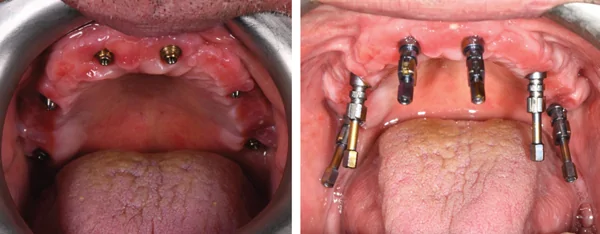
Tissues after 3 months and at new plaster impressions.

**Figure 9:**
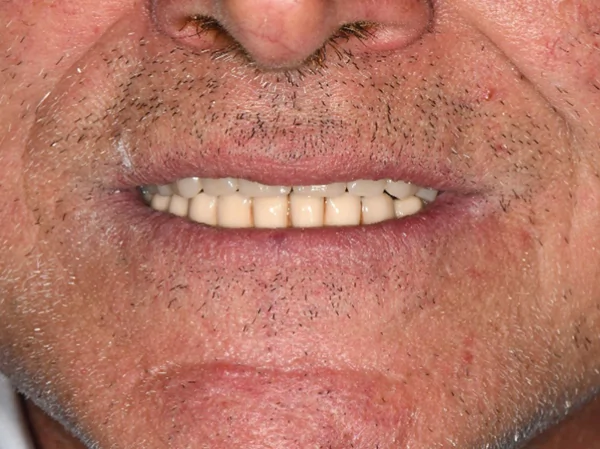
Patient exposure with the provisional prosthesis.

Three months after surgery, clinical and radiographic evaluation confirmed successful osseointegration of all implants (Figure 8). The clinical appearance with the provisional prosthesis was also evaluated at this stage (Figure 9).

At this stage, a hybrid analog–digital workflow was adopted. Conventional implant impressions were digitized using a laboratory scanner and integrated with intraoral digital records to generate a complete virtual model for CAD design.

**Figure 10:**
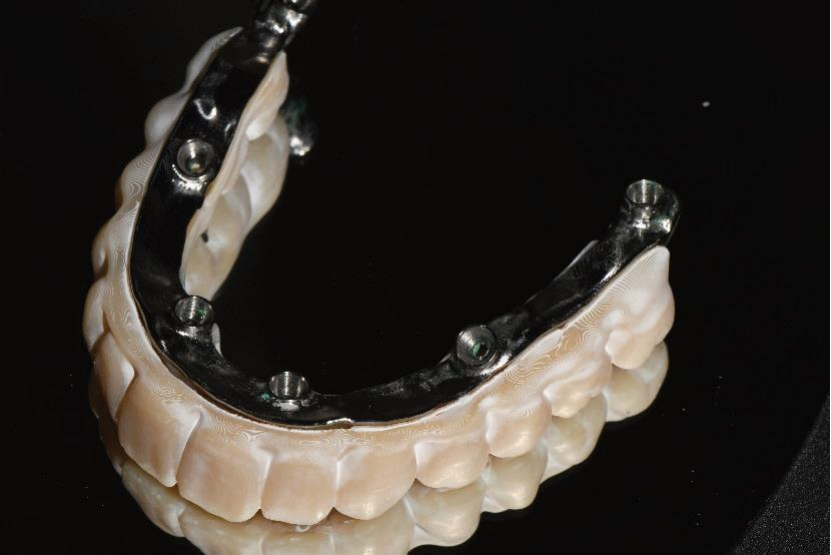
Titanium framework and resin prototype.

**Figure 11:**
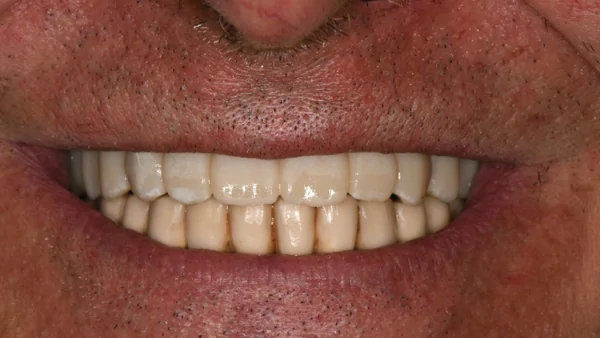
Patient exposure with the resin prototype.

A milled titanium framework was fabricated by CAD–CAM technology and veneered with composite resin. Before manufacturing the definitive restoration, a resin prototype was clinically evaluated to verify esthetics, phonetics, occlusion, and passive fit (Figure 10). This prototype was also clinically evaluated for its esthetic integration (Figure 11).

**Figure 12:**
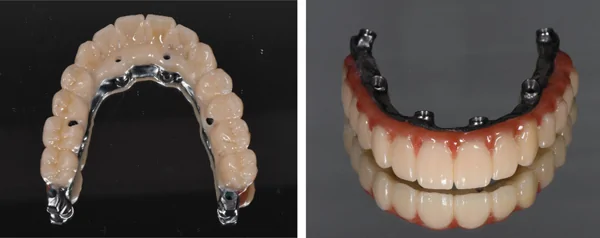
Final prosthesis view.

**Figure 13:**
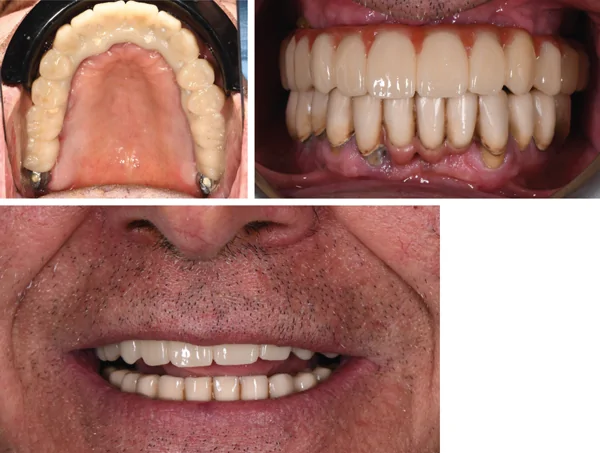
Patient aesthetic after final prosthesis delivery.

**Figure 14:**
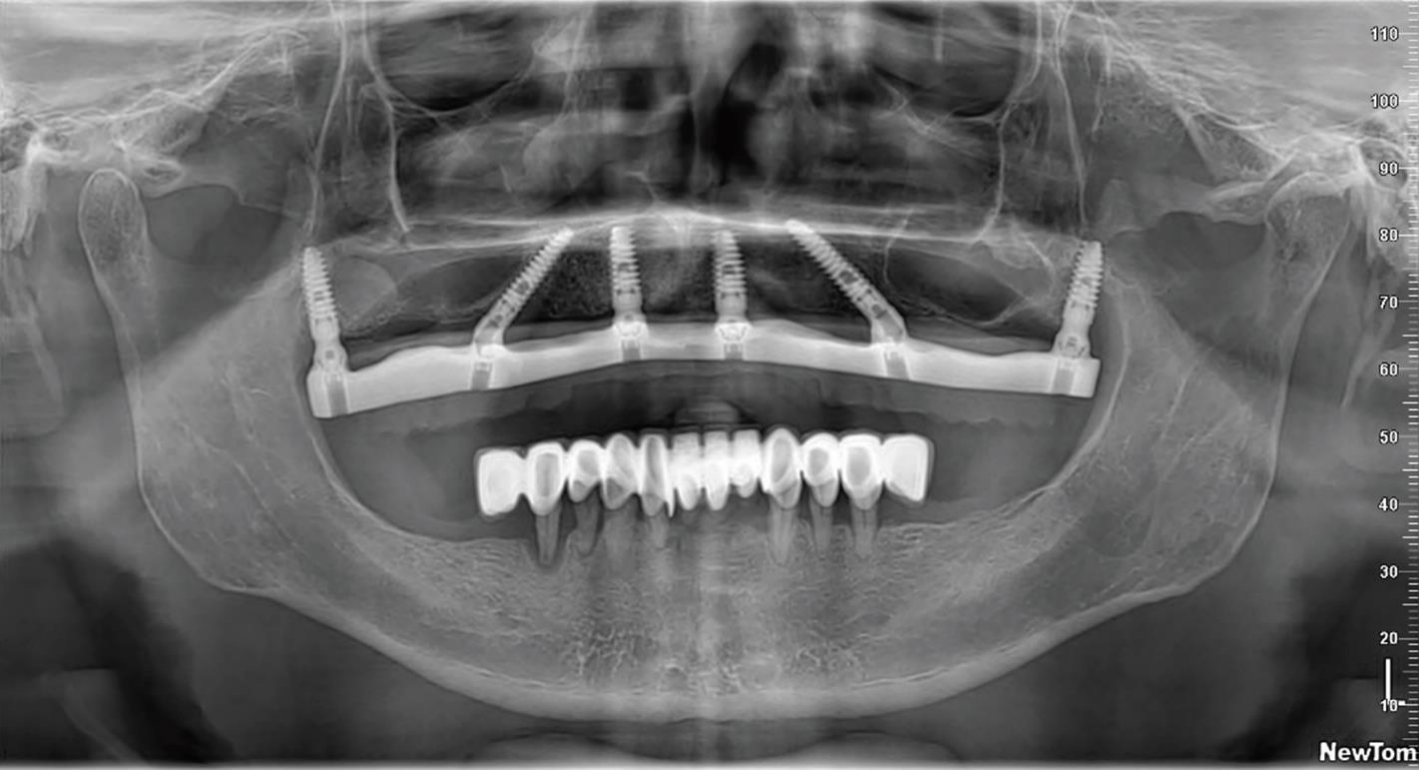
Control OPT after final prosthesis delivery.

Passive fit of the definitive framework was clinically assessed using the Sheffield test and confirmed by the one‐screw test before final delivery. Following clinical approval, the definitive screw‐retained prosthesis was delivered without requiring intraoral modifications (Figure 12). The definitive rehabilitation provided satisfactory facial and smile esthetics (Figure 13) and post‐delivery panoramic radiographic evaluation confirmed the final implant‐supported rehabilitation (Figure 14).

### 3.4. Mandibular Rehabilitation

Six months after completion of the maxillary rehabilitation, treatment of the mandibular arch was initiated following the same surgical and prosthetic philosophy.

Four implants were placed within the interforaminal region, including two axial and two tilted fixtures, all achieving insertion torque values between 35 and 50 Ncm, thus allowing immediate loading.

**Figure 15:**
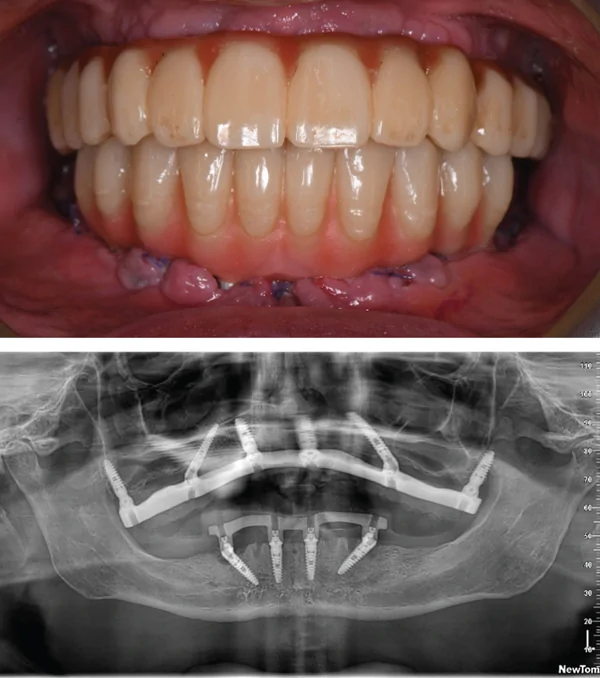
Lower immediate loading prosthesis delivered and control OPT.

**Figure 16:**
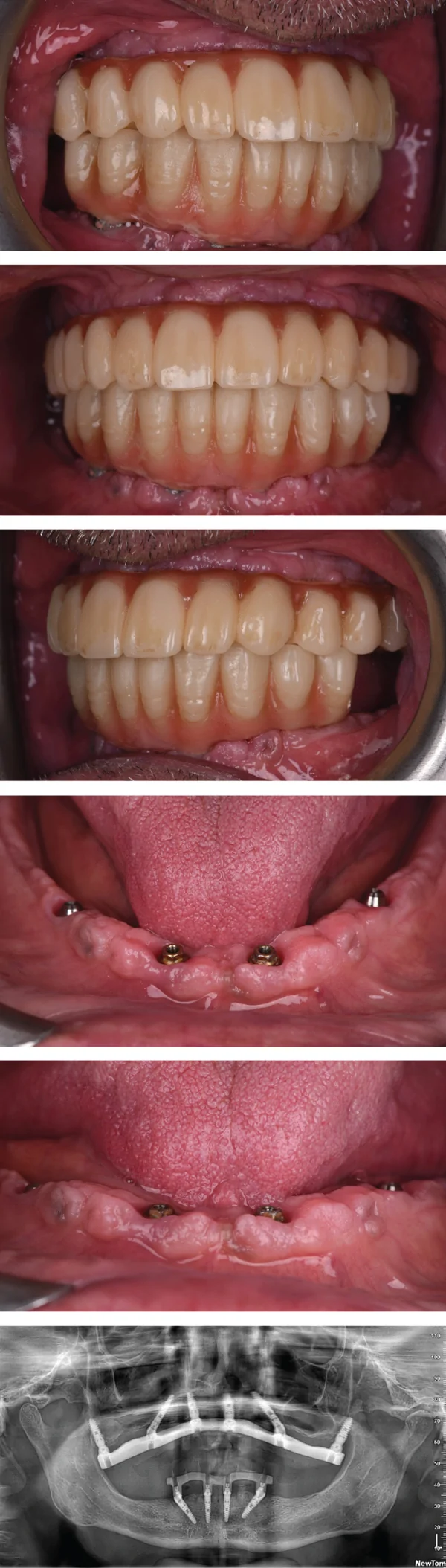
3 months osseointegration clinical and radiographical control.

**Figure 17:**
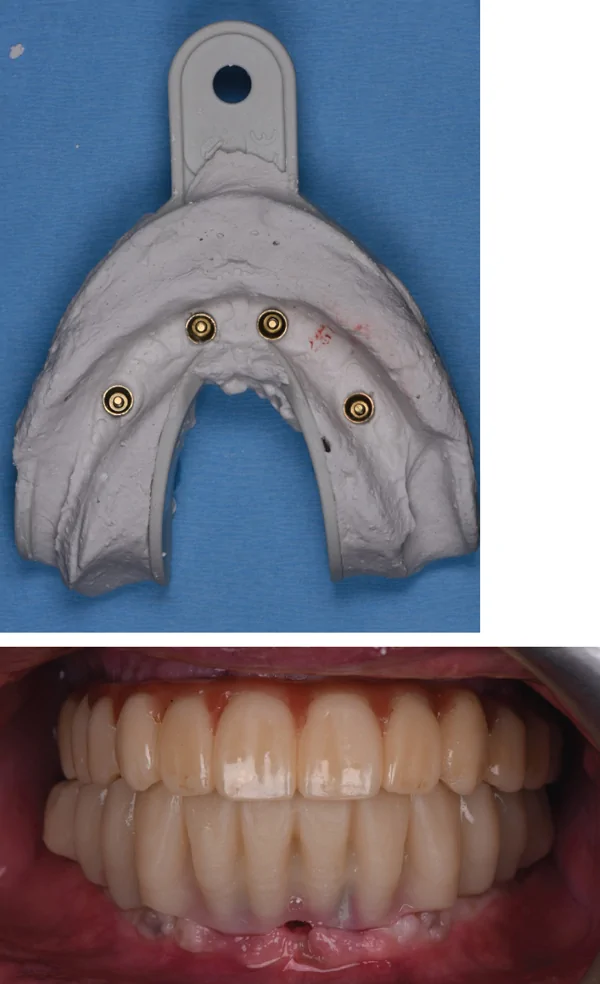
Lower definitive plaster impressions and prototype check.

Provisionalization was performed using a conventionally fabricated screw‐retained prosthesis obtained from open‐tray implant impressions (Figure 15). After a three‐month healing period, with clinical and radiographic confirmation of osseointegration (Figure 16), definitive prosthetic rehabilitation was completed using the same hybrid analog–digital workflow adopted for the maxilla. Conventional implant impressions were digitized and integrated with CAD–CAM technology to manufacture the definitive prosthesis (Figure 17).

**Figure 18:**
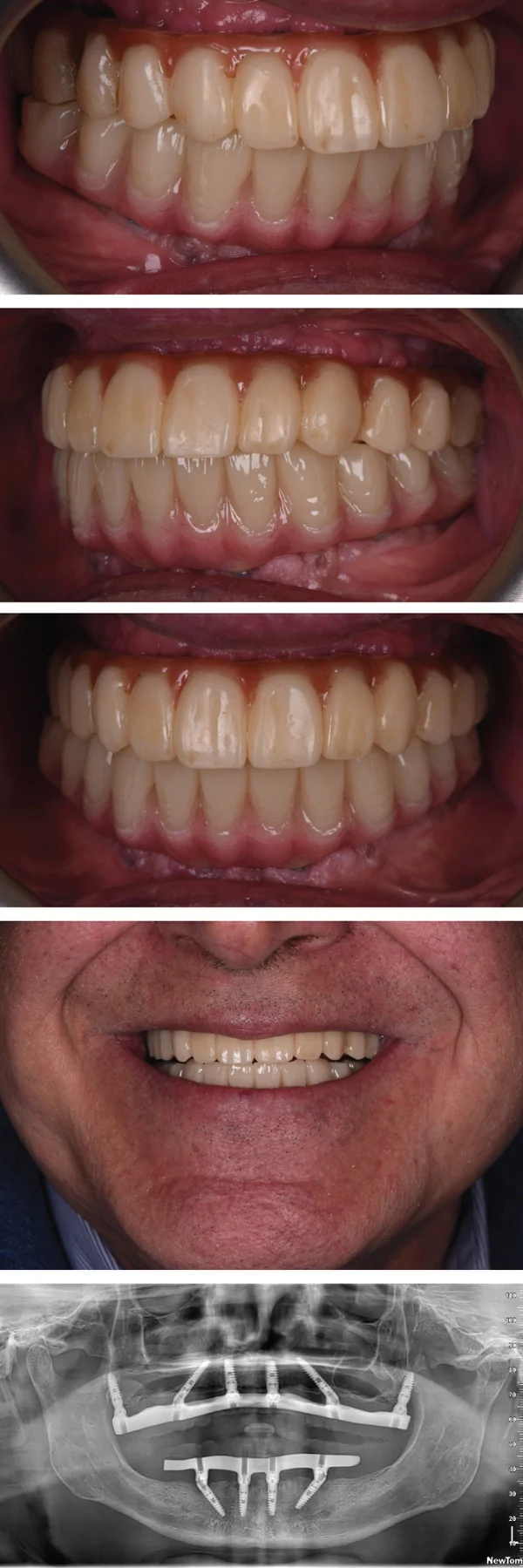
Delivery of the final prosthesis, aesthetic, and radiographic appearance.

As in the maxillary rehabilitation, passive fit was clinically verified before delivery and the definitive restoration was inserted without the need for intraoral adjustment (Figure 18).

### 3.5. Workflow Integration

For both arches, conventional and digital procedures were intentionally integrated into a single prosthetic workflow. Conventional implant impressions were considered the reference for accurate transfer of implant positions, whereas laboratory digitization and CAD–CAM technology were employed for virtual planning, framework design, and manufacturing of the definitive restorations.

Each arch was rehabilitated through six sequential clinical appointments:

• Surgical implant placement with immediate loading.
• Delivery of the immediate provisional prosthesis.
• Three‐month clinical and radiographic evaluation.
• Definitive implant impressions.
• Resin prototype try‐in.
• Delivery of the definitive CAD–CAM prosthesis.

**Figure 19:**
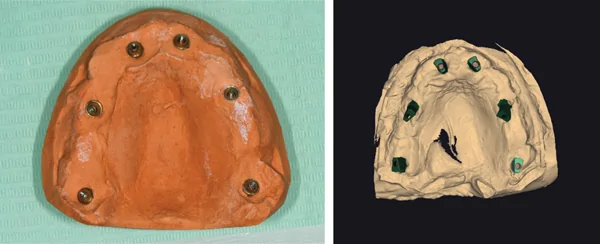
Plaster cast and digital cast view.

**Figure 20:**
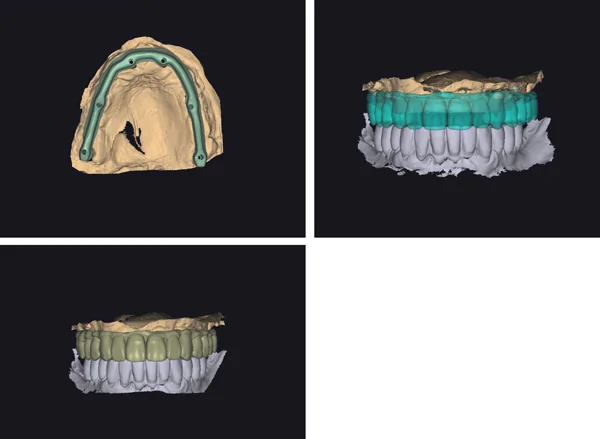
Framework and composite suprastructure project.

The chronological sequence of the diagnostic, surgical, prosthetic, and follow‐up phases is summarized in Table 1. This standardized hybrid workflow combined the predictability of conventional implant prosthodontics with the flexibility of digital technologies, providing accurate prosthetic rehabilitation while maintaining a clinically practical protocol (Figure 19, 20).

**Table 1:** Chronological timeline of diagnosis, treatment, and follow‐up.

Time	Clinical event
Baseline	Initial examination and CBCT
Day 0	Maxillary extractions and implant placement
24 h	Immediate provisional prosthesis
3 months	Definitive maxillary prosthesis
6 months	Mandibular surgery
9 months	Definitive mandibular prosthesis
24 months	Final follow‐up

### 3.6. Follow‐up and Outcomes

The postoperative healing period was uneventful in both arches. All implants achieved and maintained adequate primary stability, allowing immediate loading without early biological or mechanical complications.

Clinical and radiographic evaluation performed 3 months after maxillary surgery confirmed successful osseointegration of all six implants, including the two pterygoid implants. No implant mobility, peri‐implant radiolucency, or signs of soft tissue inflammation were observed. Likewise, the four mandibular implants demonstrated successful osseointegration 3 months after placement, with stable peri‐implant tissues and no clinical complications.

After delivery of the definitive restorations, the patient entered a structured maintenance program. During the initial healing phase, follow‐up visits were scheduled monthly and included professional plaque removal using bicarbonate and glycine powders, reinforcement of oral hygiene instructions, and clinical assessment of the peri‐implant soft tissues. Once the definitive prostheses had been delivered, the patient was enrolled in a supportive peri‐implant maintenance program with recall appointments every 3 months. The prostheses were removed every 6 months to allow professional cleaning and comprehensive peri‐implant evaluation, including probing depth assessment and monitoring of peri‐implant tissue health. Throughout the observation period, the patient was repeatedly encouraged to reduce cigarette consumption; however, no substantial reduction in smoking habits was achieved.

Radiographic follow‐up was performed using standardized panoramic radiographs obtained during the different stages of treatment. Because no clinical indications justified additional postoperative CBCT examinations, no further three‐dimensional imaging was performed, in accordance with the principles of radiation protection. Radiographic evaluation demonstrated maintenance of the peri‐implant bone peaks and stable peri‐implant bone support throughout the available observation period, without evidence of progressive bone loss.

The passive fit of both definitive prosthetic frameworks was clinically verified before delivery using the Sheffield test in combination with the one‐screw test. Both restorations exhibited complete passive seating without detectable discrepancies, and no intraoral adjustments were required before definitive insertion.

The 2‐year follow‐up refers specifically to the maxillary rehabilitation, which represented the first stage of treatment. Mandibular rehabilitation was initiated 6 months later following completion of the maxillary phase and therefore has a proportionally shorter observation period. Throughout the available follow‐up, both rehabilitations remained clinically stable.

At the latest follow‐up evaluation, stable peri‐implant soft tissues, absence of biological or mechanical complications, and preservation of prosthetic integrity were observed. No screw loosening, framework fracture, veneering material chipping, or prosthetic instability occurred.

From the patient′s perspective, treatment resulted in a marked improvement in masticatory efficiency, speech, esthetics, and overall oral comfort. The patient reported complete satisfaction with the rehabilitation and no functional complaints during the follow‐up period.

The definitive prostheses, fabricated using a hybrid analog–digital workflow, were delivered without the need for intraoral adjustments, demonstrating accurate passive fit and stable occlusion in both arches.

## 4. Discussion

Full‐arch rehabilitation of severely compromised dentitions remains one of the most demanding procedures in implant dentistry, particularly when advanced posterior maxillary atrophy limits the placement of conventionally oriented implants. In these clinical situations, the use of tilted and pterygoid implants has progressively gained acceptance as a reliable alternative to extensive bone augmentation procedures, allowing immediate loading protocols while reducing treatment time, surgical morbidity, and patient discomfort [1–6].

The present case confirms the clinical feasibility of this approach in a patient presenting several unfavorable prognostic factors, including Stage IV, Grade C periodontitis and heavy smoking. Despite these conditions, adequate primary stability was achieved for all implants, permitting immediate loading and resulting in uneventful osseointegration during the available follow‐up period. Although the successful outcome of a single case cannot be generalized, it supports previous evidence suggesting that careful patient selection, meticulous surgical execution, and strict maintenance protocols may contribute substantially to favorable implant survival even in higher risk individuals [2–6].

One of the principal aspects of this report is the adoption of a hybrid analog–digital prosthetic workflow. Digital technologies have profoundly changed contemporary implant prosthodontics, improving communication between clinicians and laboratories, facilitating CAD–CAM manufacturing, and enhancing patient comfort during several clinical procedures [2–6]. Nevertheless, the complete replacement of conventional techniques remains controversial in full‐arch implant rehabilitation.

Several investigations have demonstrated excellent accuracy for digital workflows in partially edentulous patients. However, completely edentulous arches continue to present specific challenges because of the absence of stable anatomical landmarks, increased soft tissue mobility, and cumulative stitching errors that may occur during intraoral scanning of extended implant‐supported rehabilitations [7–10]. These factors may compromise the precise transfer of implant positions and ultimately affect the passive fit of long‐span prosthetic frameworks.

Photogrammetric systems have recently been proposed to overcome many of these limitations by directly capturing implant positions with a high level of precision. Although the available evidence is encouraging, these technologies still require dedicated equipment, specific training, and considerable financial investment, which may limit their routine implementation in many private practices and clinical institutions [11–14].

Within this context, the hybrid workflow adopted in the present case should not be interpreted as an alternative dictated by technological limitations, but rather as a deliberate clinical strategy aimed at combining the strengths of both conventional and digital techniques. Conventional implant impressions continue to represent a highly reliable method for transferring implant positions in full‐arch rehabilitations, whereas laboratory digitization and CAD–CAM manufacturing allow the clinician to exploit the advantages of digital design without depending entirely on intraoral scanning accuracy [9, 15, 16].

Another noteworthy aspect concerns the use of pterygoid implants. By engaging dense cortical bone posterior to the maxillary sinus, these implants increase the posterior prosthetic support and reduce or eliminate distal cantilevers, thereby improving biomechanical load distribution across the prosthetic framework. Their use may therefore reduce the need for sinus augmentation procedures while preserving posterior occlusal support in severely resorbed maxillae [1, 2, 5].

The maintenance protocol adopted throughout follow‐up probably contributed to the favorable clinical outcome. Regular professional hygiene sessions, periodic removal of the prostheses for peri‐implant assessment, and continuous reinforcement of oral hygiene instructions were particularly important considering the patient′s history of severe periodontitis and persistent smoking habit. Although smoking cessation was repeatedly encouraged, complete discontinuation was not achieved, further emphasizing the importance of individualized supportive peri‐implant care in patients presenting persistent risk factors.

The present report also highlights an important practical consideration. In recent years, the increasing availability of digital technologies has occasionally generated the perception that fully digital workflows should represent the standard of care in every clinical situation. However, clinical decision‐making should remain guided by predictability rather than by the exclusive adoption of new technologies. The present case illustrates that a carefully planned hybrid analog–digital workflow can achieve excellent functional and prosthetic outcomes while remaining reproducible, economically sustainable, and readily applicable in routine clinical practice.

The main limitation of this report is its intrinsic nature as a single clinical case, which does not allow conclusions regarding long‐term survival or superiority over alternative workflows. Furthermore, standardized marginal bone level measurements were not available because additional postoperative three‐dimensional radiographic examinations were not ethically justified in the absence of clinical indications. Future prospective clinical studies directly comparing fully digital, hybrid, and photogrammetric workflows in full‐arch implant rehabilitation will be necessary to clarify the respective indications, limitations, and long‐term outcomes of each approach.

## 5. Conclusions

The present case demonstrates that staged full‐arch rehabilitation with tilted and pterygoid implants can be successfully combined with a hybrid analog–digital prosthetic workflow, providing predictable functional and aesthetic outcomes over the available follow‐up period.

Rather than representing a compromise between conventional and digital dentistry, the proposed workflow exploits the respective strengths of both approaches, combining the accuracy of conventional implant impressions with the versatility of CAD–CAM technologies. This strategy may represent a practical and reproducible solution for clinicians managing complex implant rehabilitations, particularly when fully digital protocols are not indicated or readily available.

Although the findings are limited by the nature of a single clinical case, they support the concept that treatment predictability depends primarily on appropriate clinical planning and execution rather than on the exclusive adoption of any specific technological workflow. Future prospective studies directly comparing hybrid and fully digital approaches will help better define the indications and long‐term performance of these treatment strategies.

## Author Contributions

**Giuseppe Alessandro Scardina:** conceptualization, validation, writing – review and editing, supervision, project administration. **Francesco Liborio:** conceptualization, methodology, investigation, data curation, writing – original draft preparation. **Fabio Massimo Sciarra:** methodology, visualization, writing – review and editing. **Enzo Maria Cumbo:** methodology, validation, writing – review and editing. **Emanuele Di Vita:** investigation, data curation, visualization, writing – review and editing. **Salvatore Nigliaccio:** data curation, writing – review and editing. **Davide Alessio Fontana:** data curation, writing – review and editing. **Pietro Messina:** validation, resources, writing – review and editing, supervision.

## Funding

Open access publishing facilitated by Universita degli Studi di Palermo, as part of the Wiley ‐ CRUI‐CARE agreement.

## Disclosure

All authors have read and approved the final version of the manuscript. Giuseppe Alessandro Scardina, as the corresponding author, had full access to all clinical data presented in this study and takes complete responsibility for the integrity of the data and the accuracy of the clinical information reported. All authors have read and agreed to the published version of the manuscript.

## Ethics Statement

According to the institutional policy, formal ethical approval was not required because this manuscript describes a single anonymized clinical case and does not constitute human subjects research. Written informed consent was obtained from the patient for both treatment and publication of the clinical information and accompanying images.

## Consent

Written informed consent was obtained from the patient for publication of this case report and all accompanying clinical and radiographic images.

## Conflicts of Interest

The authors declare no conflicts of interest.

## Data Availability

The data that support the findings of this study are available from the corresponding author upon reasonable request.
